# Ribosomal protein S3 (rpS3) secreted from various cancer cells is N-linked glycosylated

**DOI:** 10.18632/oncotarget.10180

**Published:** 2016-06-22

**Authors:** YongJoong Kim, Min Seon Lee, Hag Dong Kim, Joon Kim

**Affiliations:** ^1^ Laboratory of Biochemistry, Division of Life Sciences, Korea University, Seoul 02841, Republic of Korea

**Keywords:** glycosylation, secretion, ribosomal protein S3, ribosome

## Abstract

Ribosomal protein S3 (rpS3) is a 243 amino acid component of the 40S ribosomal small subunit. It has multiple roles in translation and extra-ribosomal functions like apoptosis and DNA repair. RpS3 is secreted only in cancer cell lines. Presently, mass spectrometry analysis revealed rpS3 to be glycosylated at the Asn165 residue. A point mutation at this residue decreased secretion of rpS3 in cancer cell lines. Secretion was also inhibited by the endoplasmic reticulum (ER)-Golgi transport inhibitor Brefeldin A and by Tunicamycin, an inhibitor of N-linked glycosylation. N-linked glycosylation of rpS3 was confirmed as necessary for rpS3 secretion into culture media via the ER-Golgi dependent pathway. RpS3 bound to Concanavalin A, a carbohydrate binding lectin protein, while treatment with peptide-N-glycosidase F shifted the secreted rpS3 to a lower molecular weight band. In addition, the N165G mutant of rpS3 displayed reduced secretion compared to the wild-type. An *in vitro* binding assay detected rpS3 homodimer formation via the N-terminal region (rpS3:1–85) and a middle region (rpS3:95–158). The results indicate that the Asn 165 residue of rpS3 is a critical site for N-linked glycosylation and passage through the ER-Golgi secretion pathway.

## INTRODUCTION

Ribosomal protein S3 (rpS3/*RPS3*/Ribosomal Protein S3) is a constituent of the 40 S ribosomal small subunit, which functions in translation. Extra-ribosomal functions include DNA repair [1, 2], apoptosis [3] and transcriptional regulation [4]. RpS3 interacts with nm23-H1, which acts as a suppressor of metastasis in certain human tumors and prevents the invasive potential in HT1080 cells [5]. Furthermore, rpS3 is overexpressed in colorectal cancer cells, suggesting that the level of rpS3 may be related to tumorigenesis [6]. A previous study showed that rpS3 was secreted into the extracellular environment in a dimeric form. The level of rpS3 secretion was prominently increased in highly malignant cells when compared to normal parent cells [7]. This suggests that secreted rpS3 may be a putative marker for malignant tumors.

About 10% of all human proteins are secretory proteins. These include cytokines, hormones, digestive enzymes and immunoglobulins [8]. Their various functions include immune defense, intercellular communication, morphogenesis, angiogenesis, apoptosis and cell differentiation [9]. Most of the secretory proteins with amino termini or internal signal sequences are targeted to the cell surface or the extracellular space. The signal sequence is recognized through a signal recognition protein (SRP) and is cleaved once the protein has crossed into the endoplasmic reticulum (ER). The newly synthesized proteins exit the ER and are coated by a cargo-containing coat protein complex II (COPII/SEC23A), targeting them for transport to the Golgi, where they are modified, processed, sorted and dispatched towards their final destination [10]. After passing through the Golgi, secretory proteins are sorted and packaged into post-Golgi transport intermediates, which move to the plasma membrane and fuse with the cell surface.

Post-translational modifications are common in eukaryotic secreted proteins. Protein glycosylation, one of the most abundant post-translational modifications in all organisms, refers to the attachment of saccharide moieties to proteins. Glycosylation participates in protein folding, interaction, stability, mobility, cell adhesion and signal transduction [11]. The glycans of secreted proteins are important for protein secretion, as they influence protein folding, provide ligands for lectin chaperones, contribute to quality control surveillance in the ER and mediate transit and selective protein targeting throughout the secretory pathway. The two major types of glycosylation are N-linked and O-linked glycosylation. Glycans are attached to polypeptide structures through amide linkages to asparagine (Asn) side chains, whereas glycosidic linkages occur with the side chains of serine/threonine (Ser/Thr), hydroxylysine or tyrosine (Tyr), with the latter involving O-glycosylation.

Approximately half of all human proteins are glycoproteins, with most containing *N-*glycan structures [12]. *N*-glycans are initially synthesized as lipid-linked oligosaccharide precursors and are transferred from the lipid-linked oligosaccharides to selected Asn residues of the polypeptides that have entered the lumen of the ER [13]. Eukaryotic organisms generally use a multi-subunit oligosaccharyltransferase on the lumenal face of the ER membrane to catalyze glycan transfer to the acceptor peptide sequences, which are comprised of an Asn-X-(Ser/Thr) tripeptide (and less frequently of Asn-X-Cysteine (Cys) and other non-standard sequons), where X can be any amino acid except for proline. Oligosaccharyltransferase facilitates the N-glycosidic linkage between the side chain amide of Asn and the oligosaccharide. Almost all glycans of glycoproteins are subject to trimming and extension as they traverse the Golgi.

The present study demonstrates that rpS3 is secreted into the cell culture medium via the ER-Golgi dependent pathway. The secretion, detected using an ELISA assay, can be used as an indicator of cancer cell malignancy *in vitro*. It is also demonstrated that N-linked glycosylation is important for rpS3 secretion and that Asn165 is the site of N-glycosylation, as confirmed by liquid chromatography-tandem mass spectrometry (LC-MS/MS) and site directed mutagenesis. Finally, rpS3 forms a homodimer through interactions of the middle and N-terminal regions.

## RESULTS

### Secretion of rpS3 is inhibited by brefeldin a, tunicamycin and monensin

rpS3 appears to be located in both the cytoplasm and nucleus. It was previously reported that rpS3 is secreted into the extracellular environment as a homodimer. Although the mechanism of secretion is not well known, the secretion of rpS3 appears to be related to the invasive malignancy of cancer cells [7].

**Figure 1:**
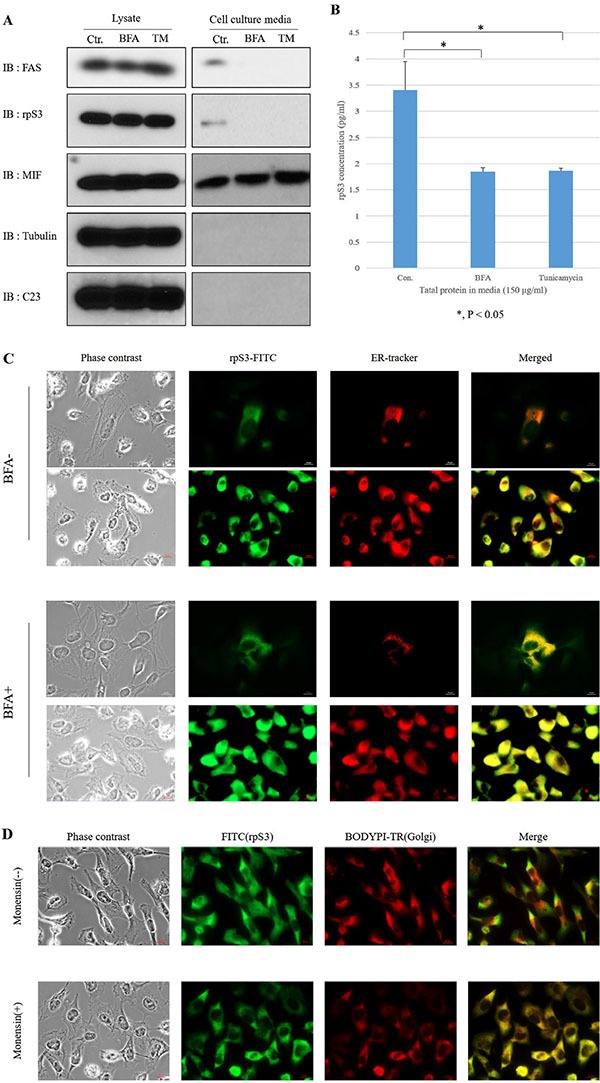
Secretion of rpS3 is inhibited by both brefeldin a (BFA) and tunicamycin (TM) **(A)** HT1080 cells were seeded at 70 ~ 80% confluency in a 150 mm diameter dish and cultured in DMEM serum-free medium with Brefeldin A (BFA) or Tunicamycin (TM) for 6 hr, after which the medium was collected and precipitated. The level of rpS3 was determined by immunoblot analysis of HT1080 cells and the medium from cultured cells. Migration inhibitory factor (MIF) was used as a marker for a non-classical export route and non-glycosylated protein. Tubulin and C23 were used as the marker of cytosol and nucleolar protein, respectively. The data were obtained from three independent replications of the experiment. **(B)** Culture medium (150 μg/ml) of HT1080 cells treated with BFA or TM were assayed for the quantity of secreted rpS3 protein by ELISA. Also, HT1080 cells were treated with BFA or Monensin, and to confirm co-localization of rpS3 with ER **(C)** or Golgi **(D)**. Error bars represent the SD of the mean for at least three independent experiments. In panels C and D: l μg/ml of BFA and TM for 6 hr and 1 μM Monensin for 8 hr.

To confirm whether the secretion of rpS3 was regulated by pathway of ER to Golgi or Golgi to ER, HT1080 cells were treated with Brefeldin A as an ER-Golgi transport inhibitor (Figure 1A and 1B) [15] or Monensin as a Golgi-ER transport inhibitor (Figure 1C and 1D) [16]. At 70 ~ 80% confluency, HT1080 cells were incubated in DMEM serum-free medium in the absence or presence of Brefeldin A or Tu. Each culture medium was collected and precipitated for immunoblot (Figure 1A and Supplementary Figure S3), ELISA (Figure 1B) and immunohistochemistry assay (Figure 1C and 1D). Brefeldin A and Monensin reduced the secretion of rpS3 (Figure 1A, 1B and Supplementary Figure S3). To further confirm the localization of the rpS3 protein in ER or Golgi, confocal immunofluorescent analysis was done using rpS3 antibody and ER-Tracker^™^ Red dye for ER or BODIPY TR dye for Golgi. Localization of rpS3 in ER (Figure 1C) and Golgi (Figure 1D) were distinctly increased in Brefeldin A and Monensin-treated cells compared with untreated cells. Fluorescence activated cell sorting (FACS) analysis using green fluorescent protein (GFP)-tagged rpS3/GFP-expressed stable cell line (Supplementary Figure S4), localization of rpS3 in ER or Golgi was increased more than 10-fold through the treatment of Brefeldin A or Monensin. These results suggest that rpS3 is secreted from HT1080 cells via the ER-Golgi and Golgi-ER pathways. To quantify ELISA assay of secreted rpS3, we performed ELISA assay with recombinant rpS3 protein (Supplementary Figure 1A and 1B).

To determine whether glycosylation is necessary for the secretion of rpS3, HT1080 cells were treated with Tunicamycin (Figure 1A and 1B) to inhibit the attachment of the precursor N-linked glycan chain to the nascent peptide, and an immunoblot assay (Figure 1A) and ELISA assay (Figure 1B) were performed. The level of rpS3 secretion decreased in the presence of Tunicamycin (Figure 1A and 1B), indicating that N-glycosylation is required for the secretion of rpS3. Tubulin and C23 were probed with the relevant antibodies to exclude the possibility that the rpS3 detected in the culture media could be derived from necrosis or rupture. C23 (*NCL*/Nucleolin), a nuclear marker, was not detected in the cell culture media. Since MIF is secreted through an unconventional secretory pathway and is not glycosylated [17], it was employed as a negative control. MIF secretion was not inhibited by Brefeldin A or Tunicamycin, and cellular apoptosis was not induced in the presence of Brefeldin A or Tunicamycin (Supplementary Figure 5A and 5B) [30]. These results suggest that the secreted rpS3 is N-glycosylated through the typical ER-Golgi route.

### Secreted rpS3 is N-glycosylated

**Figure 2:**
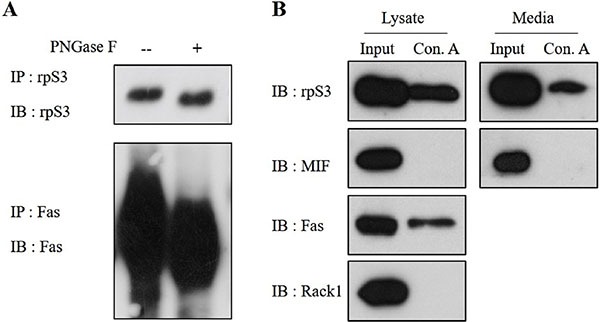
Secreted rpS3 is N-glycosylated **(A)** Concentrated HT1080 cell culture medium was not treated or was treated with PNGase F to remove N-linked glycans. The secreted rpS3 was glycosylated, as revealed by a slight downward shift of the protein band after digestion with PNGase F. **(B)** Identification of the N-linked glycans in rpS3 using a Concanavalin A (Con A) lectin binding assay. Con A isolates N-glycoproteins from cell lysates and media. Con A-bound proteins were eluted and analyzed by immunoblots. MIF, FAS and RACK1 were used as a marker of unglycosylated protein, a glycoprotein and contamination of ribosome, respectively. The data were obtained from three independent replications of the experiment.

To confirm the N-linked glycosylation of the secreted rpS3, the concentrated cell culture media were immunoprecipitated using anti-rpS3 antibody and treated with or without PNGase F, which can remove N-linked glycans. The band equivalent to the secreted rpS3 displayed a slight downward shift when digested with PNGase F, indicating that the secreted rpS3 was N-glycosylated (Figure 2A).

Lectin binding analysis was further performed to confirm N-linked glycosylation of the secreted rpS3. Con A is a lectin that recognizes alpha-linked mannose and terminal glucose residues, conferring the ability to isolate N-glycosylated proteins in cell lysates and concentrated culture media. As shown in Figure 2B, Con A bound to rpS3 in both cell lysates and media, indicating the presence of N-linked glycans. Another ribosomal protein, receptor of activated protein kinase C1 (RACK1) was not detected by Con A lectin, suggesting that ribosomes do not interact with Con A and that RACK1 is not N-glycosylated. MIF was used as a marker of unglycosylated protein, while tumor necrosis factor receptor superfamily member 6 (FAS) was employed as a marker of N-glycosylated protein. The results confirmed that secreted rpS3 is N-glycosylated.

### Identification of N-linked glycosylation sites in rpS3 by mass spectrometry analysis

To identify the sites of N-linked glycosylation in rpS3, a large amount of secreted rpS3 protein was prepared. The enriched HT1080 cell culture media was subjected to immunoprecipitation using the rpS3 antibody. The isolated rpS3 was separated by large SDS-PAGE and then stained with Coomassie brilliant blue. MS was carried out to identify which Asn residues were the sites of glycosylation. The purified rpS3 band was subjected to PNGase F digestion. Since PNGase F is an amidase, the Asn residues from which glycans were removed and became deaminated to Asp residues, resulting in an increase in the peptide mass of one1 unit. Following trypsin digestion, the deglycosylated tryptic peptides were selectively identified by LC-MS/MS. The 1 Da increase in peptide mass for each Asn-to-Asp conversion was used as a diagnostic signature to identify the glycosylated peptides [14].

The mass spectra were searched for deamination of the Asn residues using Mascot software. The ion score was −10* Log (*P*), where *P* is the probability that the observed match is a random event. Individual ion scores exceeding 4 indicate identity or extensive homology (*P* < 0.05) (Supplementary Figure S2A). Supplementary Figure S2B shows the sequence coverage map of the identified protein. The observed peptide ions accounted for 46% sequence coverage. Two (Asn22 and Asn165) of the three Asn residues in rpS3 were detected, while Asn57 peptide was not detected by MS. Therefore, we constructed N57G and NNGG as a double mutation of both Asn57 and Asn165.

The values of the molecular weight of the peptide, which can be ionized in various ways, are indicated in Supplementary Table S2. The molecular weight observed by LC-MS/MS is represented in red. Native Asn22 was detected, showing the values for the Phe^11^-Arg^40^ peptide molecular weight. The molecular weight after removal of the oligosaccharides with PNGase F is shown in Supplementary Table S2B. While the molecular mass of Asn22 was detected as 779.4046 in the glycosylated samples, it had the expected value of 780.3886 in the deglycosylated samples, which did not match. Supplementary Tables 2C and D show the molecular weight of the Phe^152^-Arg^173^ peptide with and without PNGase F treatment. The molecular mass of Asn165 was observed to be 1100.5357 in the presence of glycans, while the value of the peptide ion was replaced to 1101.5211 in the deglycosylated samples. This means the increase of 1 Da was due to the Asn-to-Asp conversion. Taken together, the LC-MS/MS data suggest that secreted rpS3 is N-glycosylated at Asn165, not Asn22. However, Asn57 remains uncertain because its fragment was not detected. Also, the result of glycosylation on Asn165 site of rpS3 protein was exactly confirmed through immunoblot assay after glycoprotein isolation with stably FLAG-rpS3 or FLAG-N165G expressed cells (Figure 4A).

### Asn165 is the site of N-glycosylation for rpS3 secretion

**Figure 3:**
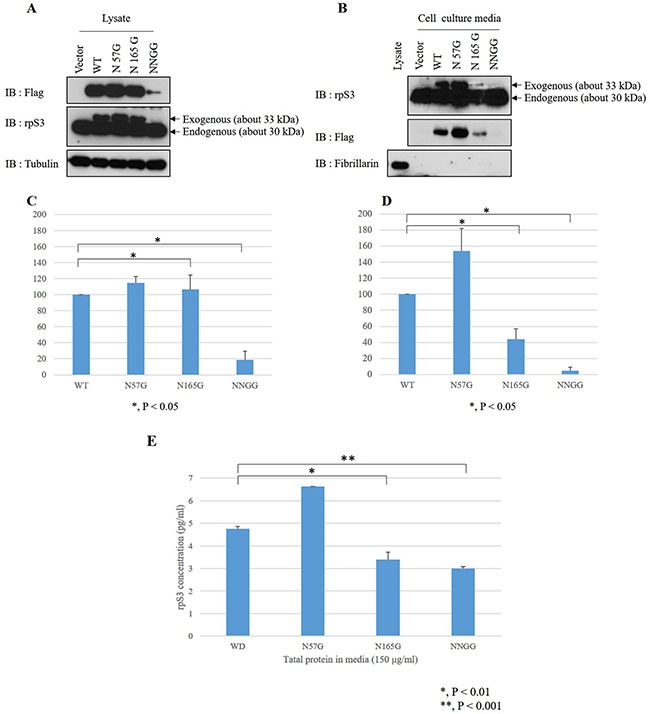
Asn165 is the site of N-glycosylation involved in rpS3 secretion N-glycosylation sites of rpS3 were mutated by site-directed mutagenesis. rpS3 wild type and N-linked glycan mutants were expressed in HT1080 cells, and cell lysates **(A)** and enriched cell culture media were subjected to immunoblotting assay with the indicated antibodies **(B)** or ELISA **(E)**. Quantitation of the expression of exogenous rpS3 in lysates **(C)** and culture media **(D)** were determined by densitometry scans of the immunoblots from panels (A) and (B). The experiments were repeated four times. Error bars represent the SD of the mean for at least three independent experiments. Fibrillarin, a nucleolar protein, was used for confirmation of cellular necrosis.

To further examine the effect of the N-glycosylation sites on the secretion of rpS3, the N-glycosylation sites of rpS3 were modified by site-directed mutagenesis. RpS3 wild-type and the mutants (N57G mutated on Asn57, N165G mutated on Asn165 and NNGG as double mutation on Asn57 and Asn165) were then stably transfected into HT1080 cells. Cell lysates and concentrated cell culture media were analyzed by immunoblotting with the antibodies indicated in Figure 3A and 3B. The expression rate of rpS3 mutants to wild type rpS3 were determined by densitometry scans. The experiments were repeated four times (Figure 3C and 3D). In the cell lysates, except in the NNGG mutant, the expression of N57G and N165G mutants were comparable to that of wild type (Figure 3A and 3C). Also, in our previous study [31], we confirmed that heat-shock protein 90 (Hsp90) regulates rpS3 stability by binding on the N- and C-termini of the rpS3 protein. But, the binding site was not determined. So, the reason for the decrease of NNGG is the possibility that the binding of rpS3 and Hsp90 protein is related with these two domains (Asn57 and Asn165) of rpS3 protein. Interestingly, as shown in Figure 3B and 3D, the N165G mutant displayed reduced secretion (approximately 44%) compared to the wild-type. However, the N57G mutant showed an even greater secretion rate (144%) than the wild-type. The NNGG mutant had significantly reduced expression and secretion, suggesting that the reduced secretion was due to degradation within the cell. Analysis of the secretion by mutants and wild type was also carried out using ELISA (Figure 3E). The results suggest that N-glycosylation of Asn165, but not Asn57, is required for the secretion of rpS3.

**Figure 4:**
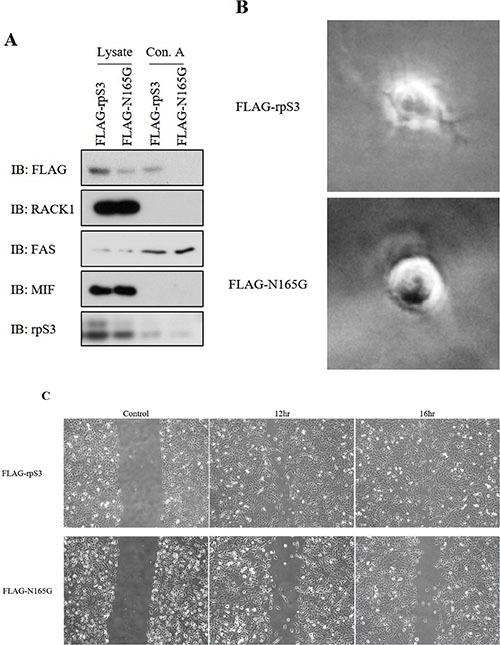
Asn165 site mutation of rpS3 is repressed invasiveness and migration of cancer **(A)** Immuno-precipitation assay with the Concanavalin A lectin were performed on stably FLAG-rpS3 or FLAG-N165G expressing HT1080 cell lines. Each protein level was confirmed by immunoblot. RACK1 was used to confirm ribosome cross-contamination. Antibody to FAS and MIF was used as a glycosylation positive and negative control, respectively. **(B)** HT1080 cancer cells that stably expressed FLAG-rpS3 or FLAG-N165G were used for 3D culture assays to identify reduction of the invasiveness phenotype. **(C)** Wound healing assays were performed on the same Ht1080 cell lines to confirm reduction of cancer cell migration by Asn165 mutation of rpS3. Following scratching, the cells were incubated for 12 hr (FLAG-rpS3) or 16 hr (FLAG-N165G). The data were obtained from three independent replications of the experiments.

### Glycosylation of rpS3 is mediated with cancer migration and the invasive phenotype

**Figure 5:**
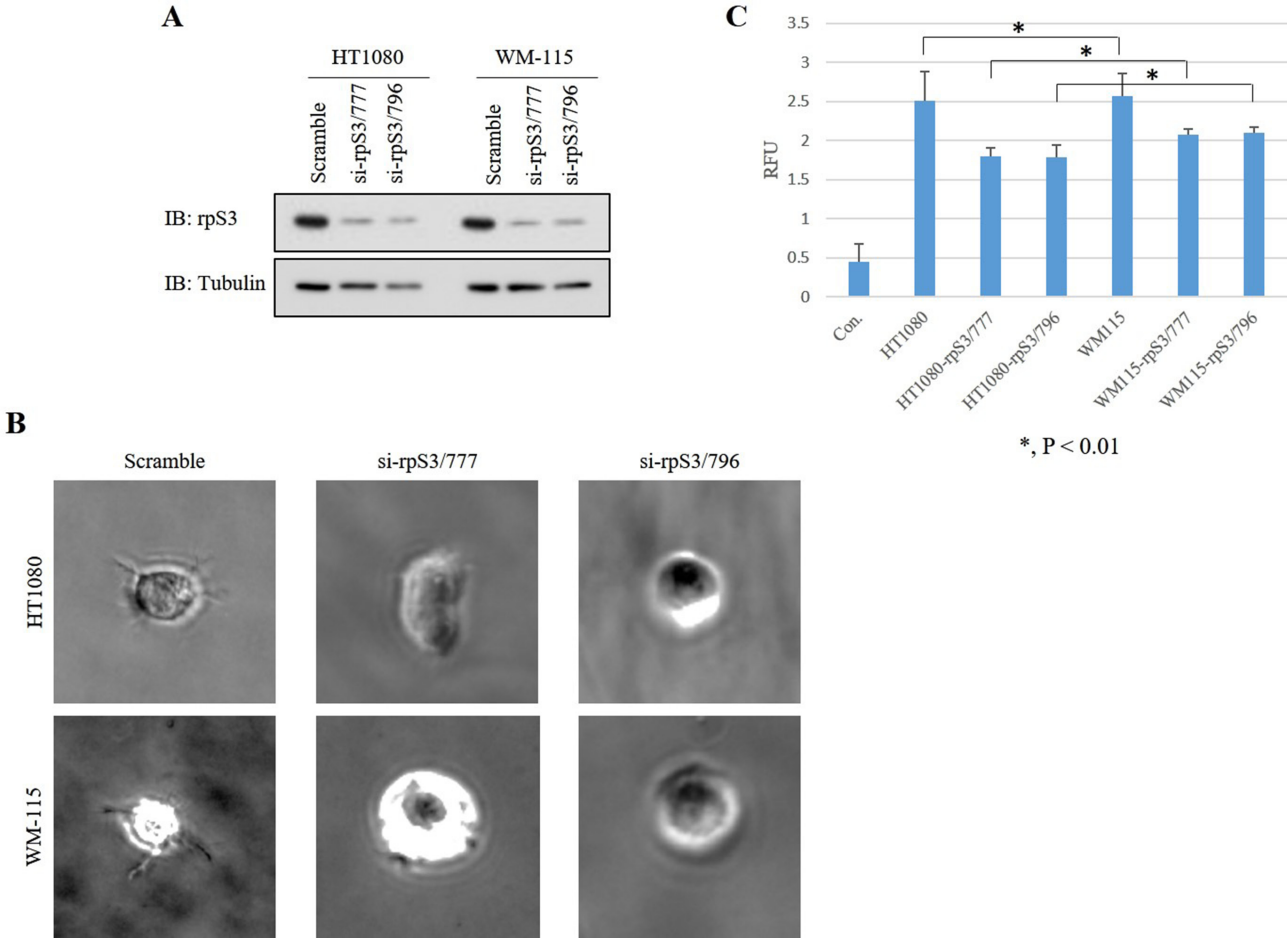
Knock-down of rpS3 decreases the invasive phenotype of malignant cancer cells **(A)** Expression of rpS3 protein in the HT1080 and WM-115 malignant cancer lines was decreased by knock-down using si-rpS3/777 and si-rpS3/796, respectively, to abrogate expression of the endogenous rpS3 gene. **(B)** Invasiveness of the phenotype was confirmed using 3D culture assay. **(C)** Quantitative analysis was performed using a dye to measure the amount of colonies. The data were obtained from three independent replications of the experiments.

We previously confirmed that the level of secreted rpS3 protein is increased in malignant cells [7]. Because secreted rpS3 requires N-glycosylation (Figure 3), we sought to confirm whether glycosylation of rpS3 is related to cancer malignancy. Migration ability and cell shape for invasion were identified using wound healing and three-dimensional (3D) culture assays (Figure 4B and 4C) with wild-type rpS3 (FLAG-rpS3) or rpS3 mutant (FLAG-N165G). After incubation of 3 days in Matrigel, the N165G mutant rpS3 showed noticeable decreases in glycosylation (Figure 4A). N165G cells had a round shape, while FLAG-rpS3 expressing cells exhibited directional alignment at their leading edges (Figure 4B). The wound healing assay showed the N165G mutation of rpS3 had reduced cancer cell migration (Figure 4C). Incubation of 7 days, as in the 3D culture assays, was performed on two malignant cancer cell lines: HT1080 fibrosarcoma cell line, and WM-115 human melanoma cell line (Figure 5B and 5C) after knock-down (Figure 5A) of endogenous rpS3 by si-RNAs (human si-rpS3/777 and si-rpS3/796). Decreased rpS3 protein levels in cells induced changes in the morphology of invasive malignant cancer cells to that of normal cells (Figure 5B). These results suggest that the glycosylation of rpS3 regulates the migration and invasive phenotype of cancer cells.

### Secretion and glycosylation of rpS3 is related with malignant phenotype of cancer cells

**Figure 6:**
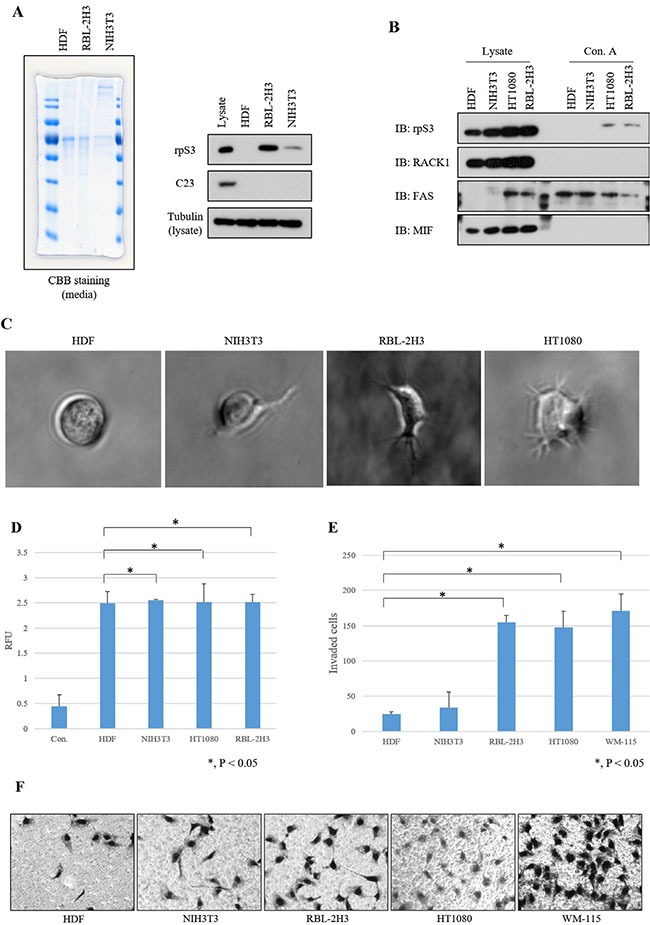
Invasive phenotype of cancer cell lines correlates with secretion level and glycosylation of rpS3 **(A)** Media-incubated human dermal fibroblasts (HDFs), HT1080 and NIH3T3 cell lines were tested for the level of rpS3 secretion. Coomasie brilliant blue (CBB) staining was performed to confirm the protein quantitation. Immunoblot assays using rpS3 and C23 anti-bodies were performed on the media of indicated cell lines. Especially, tubulin was identified for loading control with each cell line lysates. **(B)** Glycosylation status of rpS3 protein was confirmed in various cell lines. Indicated antibodies were used for confirmation of a control protein for ribosome contamination (RACK1), as a glycosylated secreted protein control (FAS) and a non-glycosylated secreted protein control (MIF). **(C)** Morphology of 3D cell invasion over 7 days. Non-invasive cells (HDF) remained as cell aggregates and did not invade the surrounding matrix. Invasive cells (RBL-2H3 and HT1080) invaded into the surrounding matrix as spindle-like protrusions. **(D)** Quantitative dye-based analysis was performed to measure the amount of colonies. The data were obtained from three independent replications of the experiments. HDFs (normal cells), NIH3T3 (immortalized cells) and RBL-2H3 and HT1080 (malignant cancer cells) were assessed using an invasion assay. **(E)** For quantitative analysis, invading cells were counted and the results were normalized. Error bars represent the SD of the mean of at least three independent experiments. **(F)** Invading cells on Matrigel were identified by microscopy examination.

To confirm that glycosylated and secreted rpS3 regulates the invasiveness of cancer cells, we identified the glycosylation status (Figure 6B) by glycoprotein isolation, cell morphology (Figure 6C and 6D) by 3D culture assay after confirmation of secretion status of rpS3 protein (Figure 6A) in a normal cell line (human dermal fibroblasts, HDFs), NIH3T3 mouse immortalized embryonic fibroblasts and a leukemia cancer cell line (RBL-2H3 rat peripheral blood fibroblasts). RpS3 secretion was increased in RBL-2H3 cells and glycosylation was increased in RBL-2H3 and HT1080 cells, but not in the immortalized cell line. Also, invasiveness of RBL-2H3, HT1080 and WM-115 cell lines, which are aggressive cancer cell lines, was dramatically increased comparing to HDF, a normal cell line and NIH3T3, a less-aggressive cell line (Figure 6E and 6F). Especially, thenormal cell line HDF, which displayed a non-invasive cell phenotype (Figure 6C and 6D) did not secrete rpS3 or glycosylated rpS3 (Figure 6A and 6B). These results suggest that glycosylated and secreted rpS3 proteins are related to invasive cell phenotypes such as malignant cancer cells.

## DISCUSSION

Our previous data demonstrated the secretion of rpS3 into the extracellular space in conjunction with tumor malignancy. However, the secretion pathway and mechanism have not yet been fully elucidated. Glycosylation is important for protein secretion, protein folding and interaction. Glycans associated with newly synthesized proteins are crucial for secretion.

Here, it was found that the secretion of rpS3 was inhibited by both Brefeldin A, an ER-Golgi transport inhibitor [15] and Monensin, a Golgi-ER transport inhibitor [16]. These indicate that rpS3 is secreted via the ER-Golgi route. In addition, Tunicamycin, an inhibitor of N-glycosylation, caused a marked decrease of rpS3 secretion, implying that N-glycans are important for the secretion of rpS3. It was also observed that the bands of secreted rpS3 slightly shifted down when digested with PNGase F, which can remove N-linked glycans. The presence of N-linked glycans in the rpS3 protein was further confirmed by the binding of rpS3 to Concanavalin A.

Most proteins destined for secretion contain an N-terminal signal sequence that mediates their translocation across the ER membrane. Many secretory proteins contain conserved N-X-V sequences (where X is any amino acid except proline), which are the non-standard sequences of N-linked glycosylation [11]. However, several proteins lacking a signal [18, 19] or conserved N-linked glycosylation site [20, 21] have also been found in the extracellular space. The signal sequence prediction server SignalP4.1 (http://www.cbs.dtu.dk/services/SignalP/) was used to predict the existence of signal sequences in rpS3. According to the prediction data, there were no significant signal sequences in rpS3, and none of the Asn residues were predicted as standard N-glycosylation sequences. Although it was not the exact consensus sequence, we revealed that rpS3 has a similar sequence (N165th-Y166th-Y167th). Tyr 167 of rpS3 and Val of non-standard sequences have the same characteristics as amino acids with a hydrophobic side chain. But, this glycosylation at the non-standard sequences was sufficient for a conventional glycosylation mechanism [11]. Also, the presence of certain carbohydrate residues on proteins can be useful markers for following their movement from the ER and through the Golgi cisternae.

For further examination of possible N-glycosylation sites, the Asn residues were investigated using MS. The LC-MS/MS data indicated that the secreted rpS3 was N-glycosylated at Asn165. The effect of site-specific glycosylation on its secretion rate was next examined using a NNGG for double mutation of Asn57 and Asn165 of rpS3. Fibrillarin was used as nucleolar protein control to confirm cell necrosis. The results indicated that the N-glycosylation of Asn165 is responsible for the secretion of rpS3. Generally, glycoproteins have a consensus motif. The N-X-V sequence in eukaryotes is one of non-standard sequences for N-glycosylation according to previous report [11]. In our study, although not the exact consensus sequence, rpS3 had the similar sequence (N165-Y166-Y167). Tyr167 of rpS3 and Val of reference have the same characteristics as amino acids with hydrophobic side chain. But, this glycosylation at the non-standard sequences appears to function for conventional glycosylation mechanism. Also, the presence of certain carbohydrate residues on proteins provides useful markers to trace their movement from the ER and through the Golgi cisternae.

RpS3 was previously reported to be secreted into the cell culture media by forming homo-dimers [7]. The binding site has not been identified. The stability maintenance of the secreted protein is an important event for its function than after. Therefore, we confirmed the binding site of homo-dimerization of secreted rpS3 (Supplementary Figure S6). To demonstrate the binding region of rpS3, an *in vitro* HIS pull-down assay was performed using GST, GST-fused rpS3, GST-rpS3 deletion mutants and His-tagged rpS3, as shown in Supplementary Figure S6A. The F85, IF159 and P mutants contained the N-terminal amino acids 1–85, amino acids 96–159 and the C-terminal amino acids 159–243, respectively. Expressions of these proteins were confirmed by Ponceau-S staining (Supplementary Figure S6B). As shown in Supplementary Figure S6C, the His-tagged rpS3 interacted with wild type (WT), F85 and IF159, but not P (rpS3; 160–243). These results suggest that rpS3 forms homodimers through the N-terminal region and a middle region.

The production of several ribosomal proteins is upregulated in several cancers [23]. Several oncogenes and tumor suppressors regulate the production of ribosomes [24–26]. In addition, rpS3 is involved in the invasion of tumor cells [27, 28]. We compared phenotype transition between HDFs transfected with wild type rpS3 and that of glycosylation defective mutant. We confirmed that HDF cells transfected with wild type rpS3 induce cell phenotype transition (Supplementary Figure S7). Our previous report confirmed that the interaction of rpS3 with nm23-H1 plays an important role in the cell invasion through the pathway of ERK phosphorylation and matrix metalloproteinase (MMP)-9 secretion [5]. Also, rpS3 regulates GLI2-mediated cancer invasion and metastasis in osteosarcoma [29]. Although the latter study concerned a specific osteosarcoma cell line, it is possible that rpS3 might play a role as a regulator in cancer invasion or metastasis. Whether the interaction with nm23-H1 correlates with the secretion level in variable cells including normal cells, cancer cells and patient samples await further studies. If these studies are confirmatory, we will know exactly whether rpS3 is secreted by the reduction of interaction with nm23-H1 in aggressive cancer cells and it will be confirmed that the rpS3 protein intracellularly functions as an inhibitor for metastasis related with nm23-H1 pathway.

## MATERIALS AND METHODS

### Cell culture

Human fibrosarcoma (HT1080), WM-115, RBL2H3 and NIH3T3 cells were cultured in Dulbecco's modified Eagle's medium (DMEM) supplemented with 10% fetal bovine serum (Hyclone, USA) and 1% penicillin streptomycin at 37°C in a humidified atmosphere of 5% CO_2_. When the cells in 150 mm diameter dishes reached 70–80% confluency, they were washed twice with Dulbecco's Phosphate-Buffered Saline (DPBS; Hyclone, USA) to remove the dead cells and were incubated with 15 mL serum-free DMEM for 16 hr. Human dermal fibroblast cells were cultured in Medium 106 with the addition of Low Serum Growth Supplement at 37°C in a humidified atmosphere of 5% CO_2_.

### Brefeldin a, tunicamycin and monensin treatment

HT1080 cells were treated with l μg/ml of Brefeldin A, l μg/ml of Tunicamycin for 6 hr or 1 μM Monensin for 8 hr, and the culture medium was collected. The medium was centrifuged at 1000 rpm for 10 min to remove cell debris, and the supernatant was carefully obtained. This procedure was repeated twice, after which the supernatants were precipitated with ethanol. In brief, three volumes of 95% ethanol (EtOH) were added to the supernatant in the presence of 0.2 M NaCl and incubated at −20°C overnight. After incubation, the mixture was centrifuged at 13,000 rpm for 10 min at 4°C, and the pellets were dried after discarding the supernatant. Each pellets was resuspended in cell lysis buffer (200 mM Tris (pH 7.5), 140 mM NaCl, 1 mM EDTA, 1 mM EGTA, 1 mM Na-glycerophosphate, 10 mM sodium fluoride, 0.25% sodium deoxycholate and 1% NP-40).

### Analysis of N-linked glycosylation of rpS3

The presence of glycans in rpS3 was determined using peptide-N-glycosidase F (PNGase F) and concanavalin A lectin. For treatment with PNGase F (NEB, USA), the supernatant was concentrated 15-fold using Amicon tubes with a molecular weight cutoff of 10 kDa (Millipore, USA). For the immunoprecipitation assay, antibody to rpS3 (2 μg) was added to the supernatant and incubated on a rocking platform for 2 hr at 4°C. Protein-A agarose beads were added and incubated for an additional 16 hr at 4°C. After incubation, the samples were washed four times with DPBS. The purified rpS3 was pre-denatured in 1 × glycoprotein denaturing buffer at 100°C for 10 min, and the denatured proteins were treated with PNGase F in a mixture with 10 × G7 buffer and 10% NP-40 at 37°C for 2 hr according to the manufacturer's recommendations. The digested proteins were analyzed by immunoblot after separation on large (21 cm × 19 cm) 12% SDS-PAGE.

Concanavalin A (Con A) was used to assess the sugar composition. Glycoproteins in the cell lysates and concentrated cell culture media were isolated using a Con A-based glycoprotein isolation kit (Thermo Scientific, USA). Con-A bound proteins were subjected to immunoblotting.

### Immunoblots

Culture media and cell lysates were separated on 12% polyacrylamide gels and transferred to PVDF membranes using a semi-dry blotting protocol. The rabbit anti-rpS3 polyclonal antibody and mouse anti-rpS3 antibody were purchased from HaimBio (Seoul, South Korea). The mouse anti-flag antibody (Thermo Scientific) was used for the detection of wild-type and mutants of flag-tagged rpS3. Antibodies to C23, fibrillarin, Fas, Rack1 and migration inhibitory factor (MIF) were supplied by Santa Cruz Biotechnology (USA).

### Identification of N-linked glycosylation sites in rpS3

To identify the sites of glycosylation in rpS3, LC-MS/MS was carried out at ProteomeTech (Seoul, Korea). First, 120 mL of medium from cultured cells were collected, and immunoprecipitation was carried out with the anti-rpS3 antibody as described above. Immunopreciptants were separated using large (21 cm × 19 cm) 12% SDS-PAGE. The gel was fixed in a mixture of 50% MeOH, 10% acetic acid and 40% H_2_O overnight and then stained with Brilliant Blue R250 (2.5 g Coomassie brilliant blue, 400 ml MeOH, 70 ml acetic acid and 530 mL H_2_O) for 2 hr. The purified rpS3 was subsequently treated with PNGase F to enzymatically remove all the N-linked glycans, yielding some oligosaccharides and a modified protein, in which the deglycosylated Asn residues were converted into Aspartic acid (Asp) residues. Following trypsin digestion, the deglycosylated tryptic peptides were selectively identified by LC-MS/MS as previously described [14].

### Site-directed mutagenesis

Construction of pcDNA3 Flag-rpS3 was carried out, and the glycosylation sites of rpS3 were modified by site-directed mutagenesis using DpnI digestion and two-step polymerase chain reaction (PCR). The primer sequences are listed in Supplementary Table S1. Mutagenesis for the N57G mutant was performed under the following conditions: 2 min at 94°C, followed by 30 cycles of 94°C for 1 min, 55°C for 30 sec and 68°C for 7 min. After DpnI digestion for 3 hr the plasmids were transformed into DH5α and the cells were grown on LB plates containing ampicillin. Two-step PCR for the N165G mutant was performed as described below. The first PCR was carried out at 94°C for 5 min, followed by 30 cycles of 94°C for 30 sec, 55°C for 30 sec and 68°C for 1 min, then polymerization at 68°C for 10 min. The PCR products were purified with a Gel Extraction Kit (Qiagen, USA) and used as the template for the second PCR after an annealing and extension step.

The conditions of the second PCR were the same as the first. The products from the second PCR were digested with XhoI and BamHI, and then extracted using phenol and chloroform. The purified PCR products were inserted into pcDNA3-Flag to generate recombinant plasmids. The recombinant plasmids were then transformed into DH5α, and the transformants were grown on LB plates containing ampicillin. Multiple clones from each plate were selected and grown, and double-stranded DNA was prepared using the plasmid Qiaprep Spin Miniprep Kit (Qiagen). All mutants were confirmed through DNA sequencing.

### Transfection

HT1080 cells were stably transfected using Lipofectamine 2000 (Invitrogen, USA) with the pcDNA3-FLAG vector, pcDNA3-FLAG-rpS3, or the plasmid containing mutant rpS3. Each stably transfected clone was selected with 600 μg/ml G418 (Calbiochem, UK) in DMEM containing 10% FBS for 4 weeks. Cell lysates and the media were collected using the method described above. The supernatants were concentrated using a Centricon centrifugal filter device with 10,000 MW (Millipore, UK). The concentrated culture media were used for immunoblotting. HT1080 cells for knock-down of endogenous rpS3 were transfected with small interfering (si) RNAs (si-rpS3/777 or si-rpS3/796) using RNAiMAX^™^ reagent (Invitrogen) according to the manufacturer's instructions. siRNAs (si-rpS3/777 or si-rpS3/796) were purchased from HaimBio (Korea). Protein expression was confirmed by Western blotting.

### *In vitro* binding assay

The complete coding region of rpS3 was inserted into pGEX_5X-1_ or pET_21a_. Glutathione-S-transferase (GST), GST-fused rpS3 (GST-rpS3), GST- rpS3 deletion mutants (F85, IF159 and P) and His-tagged rpS3 (His-rpS3) were expressed in *Escherichia coli* strain *BL21* for the GST pull-down assay. The expressed proteins were purified using glutathione–sepharose (GSH)-4B beads (Amersham Pharmacia) and Ni–NTA-agarose resin (Qiagen). The GST, GST-rpS3 and GST-rpS3 deletion mutant proteins, immobilized on the GSH-4B beads, were incubated for 16 hours at 4°C with purified His-tagged rpS3 protein. The co-precipitates were washed four times with DPBS and eluted in 2 × protein loading buffer and boiled. The eluates were then separated via 12% SDS-PAGE.

### Enzyme-linked immunosorbent assay (ELISA)

ELISA was carried out to determine the levels of rpS3 protein in the medium (extra-cellular portion). For analysis, 96-well plates were coated with monoclonal anti-rpS3 antibody (Proteintech, USA), washed four times with phosphate buffered saline (PBS) and incubated with blocking solution consisting of 1% bovine serum albumin (BSA) in PBS. Media from cultured cells were quantitated by adding 100-μL aliquots to the wells. After incubation at room temperature for 2 hr, the wells were washed five times with PBS. The samples were then incubated at room temperature for 1 hr with polyclonal anti-rpS3 antibody, washed five times with PBS, and incubated with horseradish peroxidase (HRP)-conjugated anti-rabbit IgG. After washing five times with PBS, TMB reagent (Cell Signaling Technologies, USA) was added as a substrate and then STOP buffer was added. The absorbance was measured at 450 nm with a microplate reader.

### Statistical analysis

Statistical significance was determined by Student's *t-*test. Differences were considered significant if the *p*-value was < 0.05, 0.01 or 0.001.

## SUPPLEMENTARY MATERIALS


