# Supplementary material for: Ribosomal protein S3 (rpS3) secreted from various cancer cells is N-linked glycosylated
Source: Oncotarget. 2016 Jun 22;7(49):80350–62. doi: 10.18632/oncotarget.10180 (PMC5348324; doi:10.18632/oncotarget.10180)
Supplement: Supplementary file 1 [file oncotarget-07-80350-s001.pdf]

Ribosomal protein S3 (rpS3) secreted from various cancer cells is N-linked glycosylated

Supplementary Materials

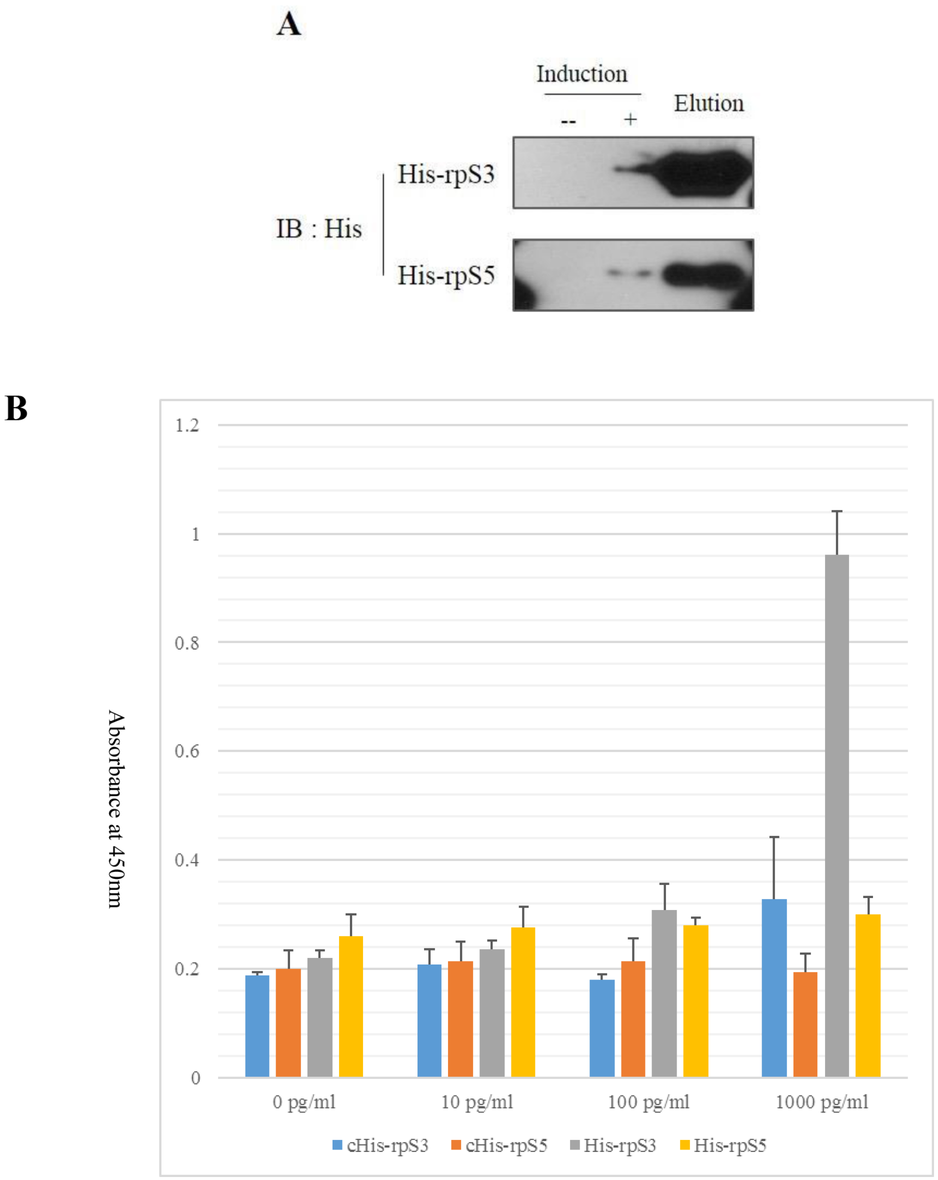

Supplementary Figure S1: His-tagged rpS3 or rpS5 inserted into a pET19b plasmid was expressed in *Escherichia coli* BL21. The expressed proteins were purified using Ni-NTA-agarose resin. The proteins were boiled in 2 × SDS sample buffer, separated by 10% SDS-PAGE, transferred to a nitrocellulose membrane, and immunoblotted using anti-His antibody **(A)** ('Induction —' : not adding IPTG, 'Induction +' : adding IPTG, 'Elution' is indicated final product), and performed ELISA assay **(B)**. Marked concentration is indicated using recombinant protein (cHis-rpS3 and cHis-rpS5 : coating ELISA plate with normal mouse IgG and then detected with rpS3 and rpS5 anti-body, His-rpS3 and His-rpS5 : coating ELISA plate with mouse monoclonal rpS3 anti-body and detecting with rpS3 and rpS5 anti-body). Error bars represent the SD of the mean of at least three independent experiments.

A

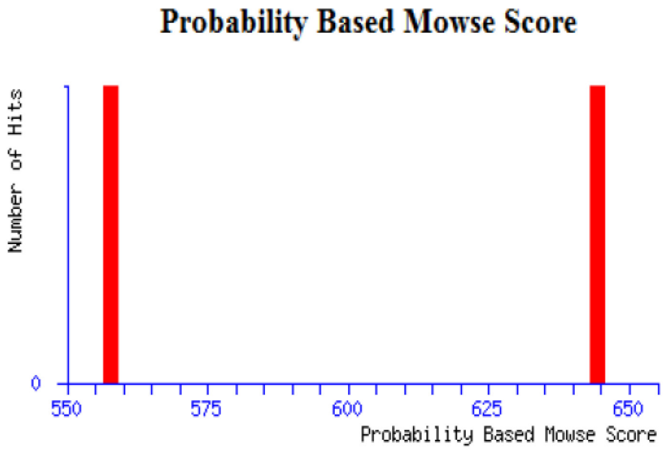

B

```
  1 MAVQISKKRK FVADGIFKAE LNEFLTRELA EDGYSGVEVR VTPTRTEIII
 51 LATRTQNVLG EKGRRIRELT AVVQKRFGFP EGSVELYAEK VATRGLCAIA
101 QAESLRYKLL GGLAVRRACY GVLRFIMESG AKGCEVVVSG KLRGQRAKSM
151 KFVDGLMIHS GDPVNYYVDT AVRHVLLRQG VLGIKVKIML PWDPTGKIGP
201 KKPLPDHVSI VEPKDEILPT TPISEQKGGK PEPPAMPQPV PTA
```

Supplementary Figure S2: Ions score is -10*Log (*P*), where *P* is the probability that the observed match is a random event. Individual ions scores exceeding 4 indicate identity or extensive homology ($P < 0.05$). Protein scores are derived from ions scores as a non-probabilistic basic for ranking protein hits (**A**). (**B**) Sequence coverage map of identified protein. Amino acids of red text correspond to those that were matched to experimental data. Asn22 and Asn165, indicated by circle and red text, were confirmed glycosylation status. Asn57 peptide (circle only) was not detected on mass spectrometry.

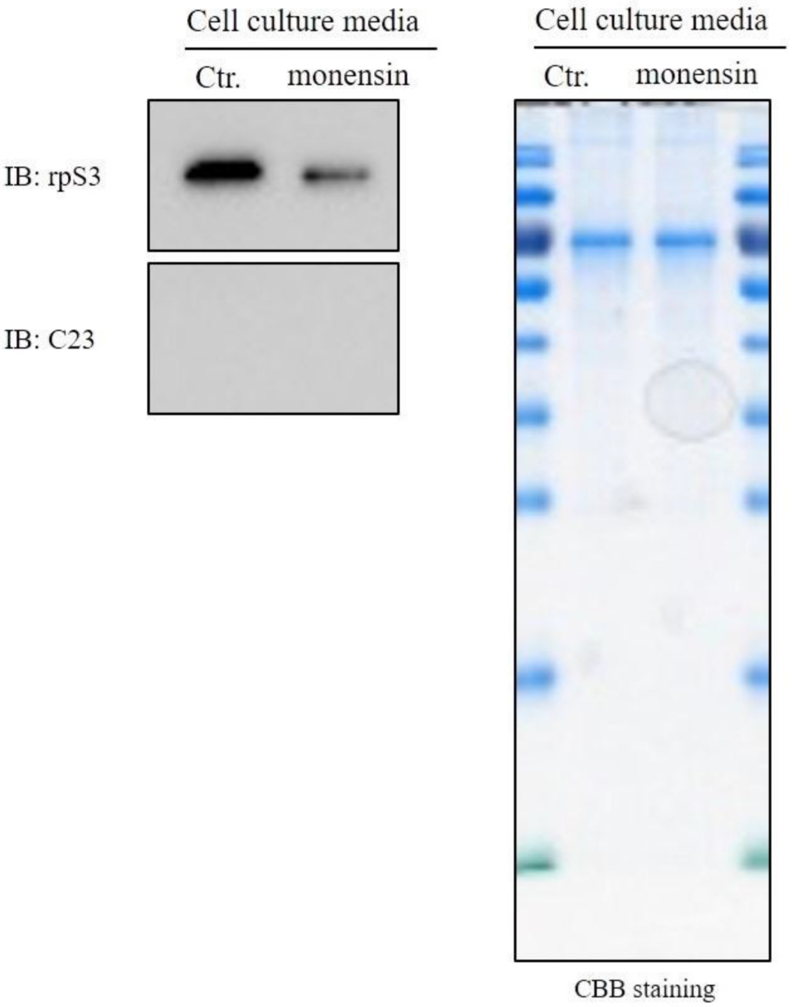

Supplementary Figure S3: RpS3 secretion was confirmed after monensin treatment. HT1080 cells were treated with Monensin to prevent protein transport from the endoplasmic reticulum to the Golgi compartment. Cell media were concentrated to perform immunoblotting (left panel) and Coomassie brilliant blue staining (right panel) after protein quantitation. C23 was used as a marker of necrosis in nucleolar protein.

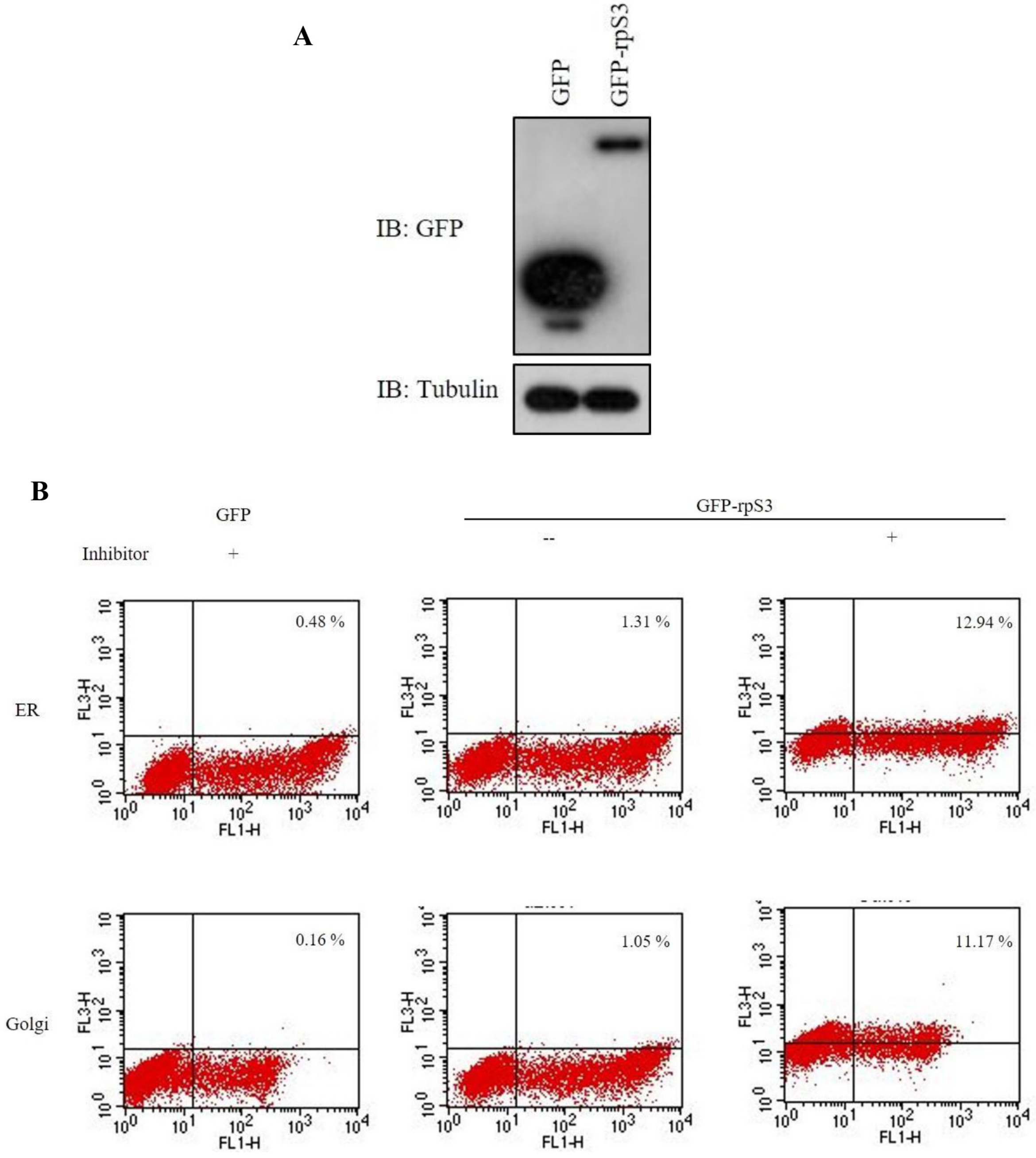

Supplementary Figure S4: RpS3 subcellular localization was confirmed by FACS analysis. HT1080 cells stably expressed GFP only or GFP-rpS3 protein were treated with/without BFA or Monensin as inhibitor of ER to Golgi or Golgi to ER, respectively. (**A**) Expression of GFP or GFP-rpS3 was confirmed by immunoblot assay using GFP anti-body. (**B**) GFP or GFP-rpS3 expressed HT1080 cells were incubated with ER-Tracker™ Red or BODYPI-TR after treatment with BFA or Monensin as inhibitor of ER or Golgi, respectively, with FACS analysis then performed. Percentage numbers denote the rate of co-localization of rps3 and ER or Golgi.

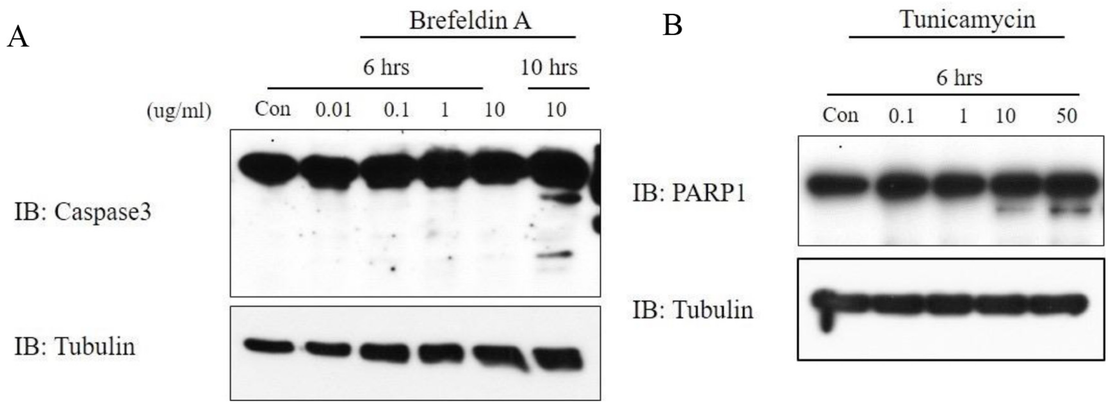

Supplementary Figure S5: Apoptosis state induced by brefeldin a or tunicamycin was confirmed on various conditions. HT1080 cells were treated with indicated concentration and incubation time of Brefeldin A or Tunicamycin were examined in an immunoblot assay. Apoptosis induced by Brefeldin A or Tunicamycin was confirmed using an immunoblot assay with caspase 3 (**A**) or PARP1 (**B**) antibody.

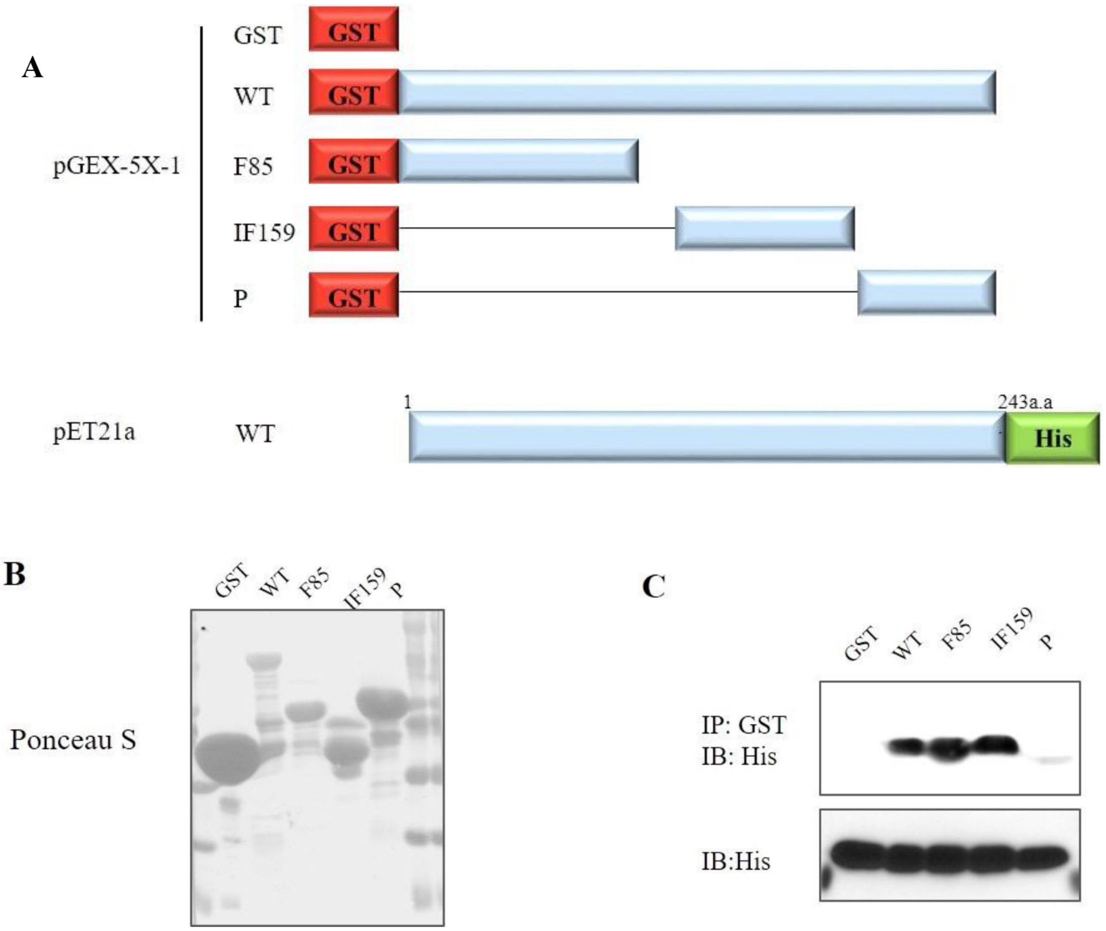

Supplementary Figure S6: RpS3 dimerization was confirmed using GST-full down assay with deletion mutants. (**A**) Schematic diagram of the wild-type and deletion mutants of rpS3. F85, IF159 and P mutants had only the N-terminal amino acids 1-85, amino acids 96-159, and the C-terminal amino acids 159-243, respectively. (**B, C**) His-tagged rpS3 protein was bound on Ni-NTA beads, then the immobilized His-rpS3 protein was incubated with purified GST, GST-S3 or GST-S3 mutants. After washing with PBS four times, the co-precipitants were eluted and separated by SDS-PAGE. Ponceau staining (B) and immunoblottting (C) were then performed.

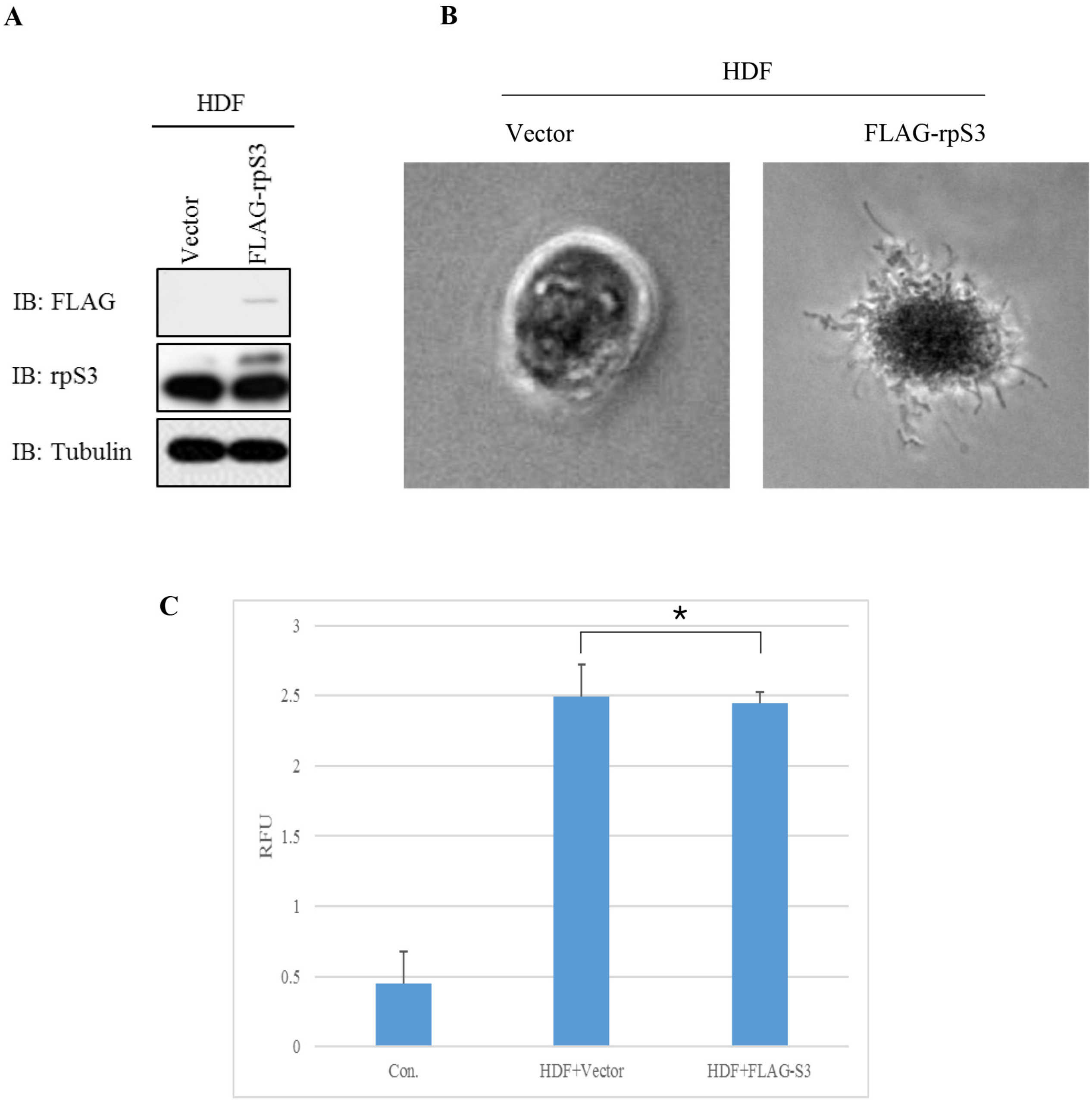

Supplementary Figure S7: RpS3 over-expression induces morphology transition of HDF cells. Vector and rpS3 wild type were expressed in HDF cells and cell lysates were subjected to immunoblotting assay with the indicated antibodies (**A**). (**B**) Morphology of 3D cell invasion over a 7 days period. Cells transfected with vector only (Vector) remain as cell aggregates and do not invade the surrounding matrix; whereas, rpS3-overexpressed cells (FLAG-rpS3) invade into the surrounding matrix as spindle-like protrusions and (**C**) Quantitative analysis was performed using a dye to measure the amount of colonies. (*n* = 3 experiments).

Supplementary Table S1: Primers used for site-directed mutagenesis

Primer	Forward primer	Reverse primer
N57G	5′TTAGCCACCAGAACACAGGG TGTTCTTGGTGAGAAGGGCCGG-3′	5′CTTCTCACCAAGAACACCCTGTG TTCTGGTGGCTAAGAT3′
N165G-1	5′AAAGGATCCTTATGCTGTGGGGACTGG3′ (Uni F)	5′AGCAGTGTCAACGTAGTAGCC AAC3′ (Mut R)
N165G-2	5AGCGGAGACCCTGTTGGCTAC3′ (Mut F)	5′TTTGGATCCTTAGATAATGATTTCTG TCCTGGT3′ (Uni R)

Uni and Mut represent universal primer and mutagenic primer, respectively.

A

Asparagine 22 of rpS3

#	b	b^{++}	b^{*}	b^{*++}	b^{0}	b^{0++}	Seq.	y	y^{++}	y^{*}	y^{*++}	y^{0}	y^{0++}	#
1	72.0444	36.5258					A							9
2	**201.0870**	101.0471			183.0764	92.0418	E	1021.5313	511.2693	1004.5047	502.7560	**1003.5207**	502.2640	8
3	314.1710	157.5892			296.1605	148.5839	L	**892.4887**	**446.7480**	875.4621	438.2347	874.4781	437.7427	7
4	**428.2140**	214.6106	411.1874	206.0973	410.2034	205.60[illegible]3	N	**779.4046**	3[illegible]0.2059	762.3781	381.6927	761.3941	381.2007	6
5	557.2566	279.1319	540.2300	270.6186	539.2460	270.1266	E	**665.3617**	333.1845	648.3351	324.6712	**647.3511**	324.1792	5
6	**704.3250**	352.6661	**687.2984**	344.1528	**686.3144**	343.6608	F	**536.3191**	268.6632	519.2926	260.1499	518.3085	259.6579	4
7	817.4090	409.2082	800.3825	**400.6949**	799.3985	400.2029	L	**389.2507**	195.1290	372.2241	186.6157	371.2401	186.1237	3
8	918.4567	459.7320	**901.4302**	451.2187	**900.4461**	450.7267	T	**276.1666**	138.5870	259.1401	130.0737	258.1561	129.5817	2
9							R	**175.1190**	88.0631	158.0924	79.5498			1

[Ions score 46]

B

Asparagine 22 of rpS3

#	b	b^{++}	b^{0}	b^{0++}	Seq.	y	y^{++}	y^{*}	y^{*++}	y^{0}	y^{0++}	#
1	72.0444	36.5258			A							9
2	**201.0870**	101.0471	183.0764	92.0418	E	1022.5153	**511.7613**	1005.4887	503.2480	**1004.5047**	502.7560	8
3	**314.1710**	157.5892	296.1605	148.5839	L	**893.4727**	**447.2400**	876.4461	438.7267	**875.4621**	438.2347	7
4	**429.1980**	215.1026	411.1874	206.0973	D	780.3886	390.6980	763.3621	382.1847	762.3781	381.6927	6
5	558.2406	279.6239	540.2300	270.6186	E	**665.3617**	333.1845	**648.3351**	324.6712	**647.3511**	324.1792	5
6	705.3090	353.1581	**687.2984**	344.1528	F	**536.3191**	**268.6632**	519.2926	260.1499	518.3085	259.6579	4
7	818.3930	409.7002	**800.3825**	**400.6949**	L	**389.2507**	195.1290	372.2241	186.6157	371.2401	186.1237	3
8	919.4407	**460.2240**	**901.4302**	451.2187	T	**276.1666**	138.5870	259.1401	130.0737	258.1561	129.5817	2
9					R	**175.1190**	88.0631	158.0924	79.5498			1

[Ions score 16]

C

Asparagine 165 of rpS3

#	b	b^{++}	b*	b^{*++}	b^0	b^{0++}	Seq.	y	y^{++}	y*	y^{*++}	y^0	y^{0++}	#
1	148.0757	74.5415					F							22
2	247.1441	124.0757					V	2337.1179	1169.0626	2320.0914	1160.5493	2319.1074	1160.0573	21
3	362.1710	181.5892			344.1605	172.5839	D	2238.0495	1119.5284	2221.0230	1111.0151	2220.0390	1110.5231	20
4	419.1925	210.0999			401.1819	201.0946	G	2123.0226	1062.0149	2105.9960	1053.5017	2105.0120	1053.0096	19
5	532.2766	266.6419			514.2660	257.6366	L	2066.0011	1033.5042	2048.9746	1024.9909	2047.9906	1024.4989	18
6	679.3120	340.1596			661.3014	331.1543	M	1952.9171	976.9622	1935.8905	968.4489	1934.9065	967.9569	17
7	792.3960	396.7016			774.3854	387.6964	I	1805.8817	903.4445	1788.8551	894.9312	1787.8711	894.4392	16
8	929.4549	465.2311			911.4444	456.2258	H	1692.7976	846.9024	1675.7711	838.3892	1674.7870	837.8972	15
9	1016.4870	508.7471			998.4764	499.7418	S	1555.7387	778.3730	1538.7121	769.8597	1537.7281	769.3677	14
10	1073.5084	537.2578			1055.4979	528.2526	G	1468.7067	734.8570	1451.6801	726.3437	1450.6961	725.8517	13
11	1188.5354	594.7713			1170.5248	585.7660	D	1411.6852	706.3462	1394.6587	697.8330	1393.6746	697.3410	12
12	1285.5881	643.2977			1267.5776	634.2924	P	1296.6583	648.8328	1279.6317	640.3195	1278.6477	639.8275	11
13	1384.6565	692.8319			1366.6460	683.8266	V	1199.6055	600.3064	1182.5790	591.7931	1181.5949	591.3011	10
14	1498.6995	749.8534	1481.6729	741.3401	1480.6889	740.848	N	1100.5371	550.7722	1083.5105	542.2589	1082.5265	541.7669	9
15	1661.7628	831.3850	1644.7362	822.8718	1643.7522	822.3797	Y	986.4942	493.7507	969.4676	485.2374	968.4836	484.7454	8
16	1824.8261	912.9167	1807.7996	904.4034	1806.8156	903.9114	Y	823.4308	412.2191	806.4043	403.7058	805.4203	403.2138	7

[Ions score 65]

D

Asparagine 165 of rpS3

#	b	b^{++}	b^0	b^{0++}	Seq.	y	y^{++}	y*	y^{*++}	y^0	y^{0++}	#
1	148.0757	74.5415			F							22
2	247.1441	124.0757			V	2322.1070	1161.5571	2305.0805	1153.0439	2304.0965	1152.5519	21
3	362.1710	181.5892	344.1605	172.5839	D	2223.0386	1112.0229	2206.0121	1103.5097	2205.0280	1103.0177	20
4	419.1925	210.0999	401.1819	201.0946	G	2108.0117	1054.5095	2090.9851	1045.9962	2090.0011	1045.5042	19
5	532.2766	266.6419	514.2660	257.6366	L	2050.9902	1025.9987	2033.9637	1017.4855	2032.9796	1016.9935	18
6	663.3170	332.1622	645.3065	323.1569	M	1937.9062	969.4567	1920.8796	960.9434	1919.8956	960.4514	17
7	776.4011	388.7042	758.3905	379.6989	I	1806.8657	903.9365	1789.8391	895.4232	1788.8551	894.9312	16
8	913.4600	457.2336	895.4494	448.2284	H	1693.7816	847.3944	1676.7551	838.8812	1675.7710	838.3892	15
9	1000.4920	500.7497	982.4815	491.7444	S	1556.7227	778.8650	1539.6962	770.3517	1538.7121	769.8597	14
10	1057.5135	529.2604	1039.5029	520.2551	G	1469.6907	735.3490	1452.6641	726.8357	1451.6801	726.3437	13
11	1172.5404	586.7739	1154.5299	577.7686	D	1412.6692	706.8382	1395.6427	698.3250	1394.6586	697.8330	12
12	1269.5932	635.3002	1251.5826	626.2950	P	1297.6423	649.3248	1280.6157	640.8115	1279.6317	640.3195	11
13	1368.6616	684.8344	1350.6510	675.8292	V	1200.5895	600.7984	1183.5630	592.2851	1182.5789	591.7931	10
14	1483.6885	742.3479	1465.6780	733.3426	D	1101.5211	551.2642	1084.4946	542.7509	1083.5105	542.2589	9
15	1646.7519	823.8796	1628.7413	814.8743	Y	986.4942	493.7507	969.4676	485.2374	968.4836	484.7454	8
16	1809.8152	905.4112	1791.8046	896.4060	Y	823.4308	412.2191	806.4043	403.7058	805.4203	403.2138	7
17	1908.8836	954.9454	1890.8731	945.9402	V	660.3675	330.6874	643.3410	322.1741	642.3569	321.6821	6

[Ions score 61]

Supplementary Table S2: Value of the molecular weight of the peptide was able to be ionized in various ways
The observed molecular weight is shown in red. LC-MS/MS peptide mass from trypsin digestion of rpS3 without (A and C) or with (B and D) PNGase F.

MATERIALS AND METHODS

Reagents and antibodies

Anti-mouse FLAG (F-3165) antibody, Brefeldin A (B6542) and Monensin (M5273) were purchased from Sigma-Aldrich (St. Louis, MO). Anti-rabbit FAS (SC-7886), MIF (FL-115), anti-mouse C23 (SC-8031), anti-mouse RACK1 (SC-17754) and anti-mouse His-probe (sc-8036) were obtained from Santa Cruz Biotechnology (Santa Cruz, CA). Anti-human rpS3 antibody was purchased from HaimBio (Seoul, South Korea). ECL reagents were purchased from Pierce (34078; Rockford, IL). Lipofectamine was purchased from Invitrogen (Carlsbad, CA).

3D culture and wound healing assay

To perform 3D culture assays, 24-well plates were pre-coated with 300 μL of matrigel (BD Biosciences, Erembodegem, Belgium). The plates were placed in a 37°C incubator for 15 min, allowing the Matrigel to polymerize. Medium (600 μL) including the cells (0.1 × 104) and 2% Matrigel were added to the plate and then incubated at 37°C in an incubator for indicated time. Quantitative analysis was performed using CytoSelect 384-well Cell Transformation Assay (CBA-145, CELL BIOLABS, USA) according to the manufacturer's instructions. To perform the wound healing assay, monolayers of various cell lines (NIH3T3, RBL-2H3, HT1080 and WM-115 human dermal fibroblasts) grown to 90% confluence were scratched using a 200 μL tip and the medium was replaced with fresh growth medium. After allowing migration for 16 h, the cells were fixed with 3.7% paraformaldehyde for 30 min, followed by staining with 1% crystal violet in 10% ethanol for 30 min.

Immunocytochemistry and FACS analysis

For ER or Golgi staining, HT1080 cells were incubated for 30 min with pre-warmed growth medium containing 200 pM ER-Tracker™ Red dye (E34250; Molecular Probes, Eugene, OR, USA) for ER or 5 μM BODIPY TR (B-34400, Molecular Probes) for Golgi. The cells were then fixed with 3.7% formaldehyde for 15 min at 37°C and permeabilized with 0.1% Triton X-100 in phosphate buffered saline (PBS) for 10 min at room temperature. The cells were incubated with rabbit anti-rpS3 polyclonal antibody and then fluorescein isothiocyanate-conjugated secondary antibodies. Fluorescent cells were examined using a LSM 5 Exciter confocal laser scanning microscope (Carl Zeiss, Jena, Germany). To perform FACS analysis, HT1080 cells that stably expressed GFP or GFP-rpS3 were stained with the aforementioned staining dye. The harvested cells were washed once with PBS and fluorescence was measured by FACS analysis.

Invasion assay

Invasion assays were performed using Boyden chambers equipped with Transwell® permeable supports (Corning, Corning, NY, USA). Cells (2 × 104) in 200 μL of medium from the aforementioned fibroblast lines were re-seeded onto the upper surface of Matrigel (BD Biosciences, Erembodegem, Belgium)-coated filters in Boyden chambers. The lower chambers were filled with 850 μL medium. After 20 h of incubation, the cells that moved through to the lower surface of the filters were fixed and stained using a Diff-Quick stain kit (Polyscience, Warrington, PA, USA). The stained cells were counted using a microscope.

REFERENCES

1. Kim Y, Kim HD, Youn B, Park YG, Kim J. Ribosomal protein S3 is secreted as a homodimer in cancer cells. Biochemical and biophysical research communications. 2013; 441:805–808
